# Adaptive questionnaires for facilitating patient data entry in clinical decision support systems: methods and application to STOPP/START v2

**DOI:** 10.1186/s12911-024-02742-6

**Published:** 2024-11-05

**Authors:** Jean-Baptiste Lamy, Abdelmalek Mouazer, Romain Léguillon, Romain Lelong, Stéfan Darmoni, Karima Sedki, Sophie Dubois, Hector Falcoff

**Affiliations:** 1 INSERM, Sorbonne Universite, Universite Sorbonne Paris Nord, Laboratory of Medical Informatics and Knowledge Engineering in e-Health, LIMICS, 15 rue de l’école de médecine, Paris, 75006 France; 2 Department of Biomedical Informatics, Rouen University Hospital, Rouen, 76000 France; 3 SFTG Recherche (Société de Formation Thérapeutique du Généraliste), Paris, 75013 France

**Keywords:** Adaptive questionnaire, Clinical decision support systems, Polypharmacy management, Medication review, Patient data entry, STOPP/START v2

## Abstract

Clinical decision support systems are software tools that help clinicians to make medical decisions. However, their acceptance by clinicians is usually rather low. A known problem is that they often require clinicians to manually enter a lot of patient data, which is long and tedious. Existing solutions, such as the automatic data extraction from electronic health record, are not fully satisfying, because of low data quality and availability. In practice, many systems still include long questionnaire for data entry. In this paper, we propose an original solution to simplify patient data entry, using an *adaptive* questionnaire, *i.e.* a questionnaire that evolves during user interaction, showing or hiding questions dynamically. Considering a rule-based decision support systems, we designed methods for determining the relationships between rules and translating the system’s clinical rules into display rules that determine the items to show in the questionnaire, and methods for determining the optimal order of priority among the items in the questionnaire. We applied this approach to a decision support system implementing STOPP/START v2, a guideline for managing polypharmacy. We show that it permits reducing by about two thirds the number of clinical conditions displayed in the questionnaire, both on clinical cases and real patient data. Presented to clinicians during focus group sessions, the adaptive questionnaire was found “pretty easy to use”. In the future, this approach could be applied to other guidelines, and adapted for data entry by patients.

**Supplementary Information:**

The online version contains supplementary material available at 10.1186/s12911-024-02742-6.

## Introduction

Clinical decision support systems (CDSS) [1, 2] are software tools aimed at helping clinicians to make medical decisions, typically regarding diagnosis or therapy. Many CDSS have been proposed for chronic diseases [3], most of them implementing the paper clinical practice guidelines produced by learned societies. CDSS have the potential for improving healthcare. Meta-analysis [4, 5] showed that CDSS are effective at improving healthcare processes, despite their clinical and economic impacts are more difficult to assess.

In the ABiMed research project [6, 7], our general objective is to design a CDSS for medication reviews. A medication review is a structured patient interview carried out by the community pharmacist with the aim of optimizing patient care, for aged patients with polypharmacy (*i.e.* consuming five drugs or more). Several CDSS have been proposed for medication reviews [8–10], most of them being based on the implementation of guidelines.

However, clinicians acceptance with regard to CDSS is often rather low, and many reasons have been identified [11–13], including low computer literacy, low trust, reduction of professional autonomy, poor integration within the workflow, lack of time and the need for a lot of patient data entry. Here, we will focus on the latter problem: for running the CDSS and obtaining recommendations, a clinician has to enter the clinical data of his/her patient, which is a tedious and time-consuming task. It has been shown that patient data entry contributes to physician burnout [14], but also that a lot of data entry can be associated with entry errors, leading to wrong decisions [15].

Several solutions have been proposed for helping with patient data entry. In particular, semantic interoperability [16] permits the CDSS to reuse patient data previously entered elsewhere, typically in the electronic health record (EHR). For instance, in France, EHR exist and are usually based on the ICD10 terminology (International Classification of Diseases, release 10). However, many patient conditions are still entered in EHR as free-text, and not coded using a medical terminology, impairing its reuse. This is especially true in primary care, where EHR are less structured and generalized than at hospital, and patient data is often spread over several EHR, owned by the GP and physicians of various medical specialties. Some CDSS may require data that is commonly not coded in EHR, either because it is too specific, or judged too trivial by the clinicians (e.g. all GPs do not enter symptoms like “cough” in the EHR). Another option, natural language processing [17], allows the automatic coding of free-text, but almost never achieve a 100% accuracy. Moreover, various physicians may use the same medical terminology in a different manner, resulting in different terms for the same patient [18]. To conclude, none of these solutions is perfect, and clinicians still have to check the patient data used by the CDSS and to enter the missing data, if any.

This problem of data entry is particularly pregnant in the context of medication review. In fact, community pharmacists do not have access to the GP or hospital EHR. Existing CDSS for medication review either (1) target hospital pharmacists or physicians [19], despite medication review is normally performed outside hospital by community pharmacists, (2) require that the pharmacist enter all patient data manually [20], or (3) implement only simple rules that can be executed without patient data other than the drug order [21] (*i.e.* no patient conditions). Consequently, for our CDSS, we have to rely on manual data entry.

In practice, two approaches are commonly used for patient data entry in CDSS. The first approach consists of a list of patient conditions restricted to the conditions specifically relevant for the CDSS, associated with checkboxes. We used this approach in the past in the ASTI project [22]. However, the list quickly becomes long as the number of parameters considered by the CDSS increases. The second approach consists of asking the clinicians to enter all patient conditions, e.g. using a terminology such as ICD10. But this is even more tedious.

However, in various domains, adaptive questionnaires have been used to facilitate data entry. A questionnaire is adaptive “when its question sequence is dynamically driven by the answers of the taker” [23], i.e. the questionnaire evolves during user interaction, typically by showing or hiding questions.

The objective of this paper is to describe an adaptive questionnaire for helping pharmacists with patient data entry in a rule-based CDSS for medication reviews. Since drug prescriptions are usually entered and coded in computerized provider order entry (CPOE) or pharmacy software, we will focus on the entry of patient clinical conditions. As a basic example, if a rule triggers if the patient has both hypertension and type 2 diabetes, an adaptive questionnaire may hide the “type 2 diabetes” question until the “hypertension” question is answered positively (or *vice versa*). Our approach consists in determining the relationships between rules and automatically translating the CDSS rule base into a new set of rules that determine whether each patient condition is shown, and the heuristic ordering of patient conditions for finding the best priority order between them (e.g. should we ask first for hypertension or for type 2 diabetes in the example above?). The approach also includes a dedicated user interface for the questionnaire. We evaluated our system in terms of reduction of the number of patient conditions asked, both on clinical cases and real patient data, and in terms of clinicians opinion during focus group sessions.

## Related works

Adaptive questionnaires should be distinguished from *dynamic forms*. A dynamic form is a questionnaire that is dynamically generated from a knowledge source [24]. But contrary to an adaptive questionnaire, a dynamic form does not change during the user interaction. F Sadki et al. [25] proposed an example of dynamic forms, through a web application that generates a semantically structured web form from an ontology.

From our literature review, we can distinguish various categories of adaptive questionnaires. An adaptive questionnaire can be *ordered*, when the questions must be answered in a given order and new questions only appear after the last question answered, or *unordered*, when questions can be answered in any order and new questions may appear anywhere in the questionnaire (after or before the last answered question). An adaptive questionnaire can be *exhaustive*, when all relevant questions are shown (e.g. pregnancy status is relevant for a female patient but not for a male patient), or *inexhaustive*, when only the most important questions are shown, the importance of the questions being evaluated with regards to their impact on the decision, usually according to some heuristic rule (e.g. pregnancy status may have a low impact on the decision for a particular female patient, and thus not shown).

Several ordered inexhaustive adaptive questionnaires were proposed for assessing learning style in education. E Mwamikazi et al. [26] proposed a system that classifies students in Myers-Briggs Type accurately while asking 81% fewer questions than state-of-the-art systems. The system relies on a question sorting algorithm that takes into account the discriminative power of the questions with regards to the Myers-Briggs Type class. A Ortigosa et al. [27] proposed AH-questionnaire, a system based on the C4.5 algorithm and decision trees, for reducing the number of questions asked for determining the Felder-Silverman’s Learning Style Model. USHER [28] combines a dynamic form with an ordered inexhaustive adaptive questionnaire. The system learns a probabilistic model over the questions for ordering them. In addition, USHER can also re-ask questions that have a high probability of being associated with an erroneous answer. ADAPQUEST [23] is a Java tool for the development of ordered inexhaustive adaptive questionnaires, based on Bayesian networks. The tool has been applied to the diagnosis of mental disorders. C Paduraru et al. proposed the use of AI agents generating an adaptive questionnaire for profiling people [29].

Several systems have been proposed for helping patients to enter their medical data. DQueST [30] proposes an unordered inexhaustive adaptive questionnaire for helping patients to find clinical trials for which they are eligible. The system starts like a free-text search, as usual search engines. Then, it ranks questions and identify the most informative ones. The approach is unordered, the user being able to choose what type of question he/she will answer next (e.g. a question on diagnosis or prescribed treatment). RC Gibson et al. [31] proposed an ontology-based ordered exhaustive adaptive questionnaire in order to help patients with learning disabilities to report their symptoms. The questionnaire adapts itself according to the patient answer, but also according to his/her disabilities. X Kortum et al. [32] proposed an ordered inexhaustive adaptive questionnaire for self-diagnosis. The authors rely on machine learning for selecting and ordering questions. PC Sherimon et al. [33] proposed an ordered exhaustive adaptive questionnaire for helping patients to enter medical data. The system is associated with a CDSS for diabetes.

Chronic-pharma [34] includes the LESS-CHRON module, aimed at helping to deprescribe drugs. It proposes an ordered exhaustive adaptive questionnaire for entering patient drug order and main conditions. The questionnaire is structured on three levels: first, the clinician select the anatomic groups of the drugs taken by the patient (e.g. cardiovascular), then the drug classes (e.g. antihypertensives), and finally the prescription characteristics (e.g. treatment duration) and relevant patient conditions (e.g. systolic blood pressure<160 mmHg). At levels 2 and 3, only the items corresponding to the items selected at the previous level are shown.

## Methods

In this work, we opted for an *unordered exhaustive* adaptive questionnaire. We opted for an exhaustive approach because all relevant questions have to be asked in a guideline-based CDSS. We opted for an unordered approach, despite most existing approaches are ordered as seen above, because clinicians usually expect clinical conditions to be organized by anatomy (e.g. cardiac, renal...) and/or by etiology (e.g. infectious diseases), as they are in medical terminologies such as ICD10. On the contrary, in an ordered adaptive questionnaire, clinical conditions would have been ordered according to some heuristic criteria, which is counterintuitive for clinicians.

### Methods for the adaptive questionnaire

#### Brief presentation of our CDSS for medication reviews

Our CDSS implements the STOPP/START v2 clinical guideline for medication reviews [35]. STOPP/START v2 includes 114 recommendations for elderly patients with polypharmacy, with both recommendations for stopping current inappropriate drug treatment (STOPP) and for starting new prescriptions (START) when missing (e.g. to prevent or control adverse events, such as starting a laxative for patients taking opioid). The CDSS was implemented in Python 3 with the Owlready 2 ontology-oriented programming module [36] for Python. Clinical rules were expressed in a high-level language that is automatically translated into SPARQL queries. Clinical conditions were coded using ICD10, drugs with ATC (Anatomical Therapeutical Chemical classification of drugs) and lab test results sith LOINC (Logical Observation Identifiers Names & Codes). Our CDSS uses the Theriaque French drug database for accessing drug properties. For more details on the implementation of STOPP/START, please refer to the coded algorithms proposed by CJA Huibers et al. [37] and the previous publication on our rule-based system [38]. The next subsection describes the rule format of our system.

#### Clinical rule formalization

Let us consider $C=\left\{ C_{1},C_{2},...,C_{n}\right\}$, a set of *n* clinical conditions, $D=\left\{ D_{1},D_{2},...\right\}$, a set of non-clinical conditions (e.g. prescribed drugs), and $R=\left\{ R_{1},R_{2},...,R_{m}\right\}$, a set of *m* clinical rules of the following form:

$$\begin{aligned}\begin{array}{cl} & C_{1}^{p}\wedge C_{2}^{p}\wedge ...\wedge D_{1}^{p}\wedge D_{2}^{p}\wedge ...\\ \wedge & \lnot C_{1}^{a}\wedge \lnot C_{2}^{a}\wedge ...\wedge \lnot D_{1}^{a}\wedge \lnot D_{2}^{a}\wedge ...\\ \wedge & \left( C_{1}^{u1}\vee C_{2}^{u1}\vee ...\vee D_{1}^{u1}\vee D_{2}^{u1}\vee ...\right) \\ \wedge & \left( C_{1}^{u2}\vee C_{2}^{u2}\vee ...\vee D_{1}^{u2}\vee D_{2}^{u2}\vee ...\right) \wedge ...\,\rightarrow A \end{array} \end{aligned}$$

where $C^{p}=\left\{ C_{1}^{p},C_{2}^{p},...\right\}$ and $D^{p}=\left\{ D_{1}^{p},D_{2}^{p},...\right\}$ are the clinical and non-clinical conditions that must be present in the patient for triggering the rule, respectively, $C^{a}=\left\{ C_{1}^{a},C_{2}^{a},...\right\}$ and $D^{a}=\left\{ D_{1}^{a},D_{2}^{a},...\right\}$ are the clinical and non-clinical conditions that must be absent, $C^{u1}=\left\{ C_{1}^{u1},C_{2}^{u1},...\right\}$ and $D^{u1}=\left\{ D_{1}^{u1},D_{2}^{u1},...\right\}$ are the clinical and non-clinical conditions that are members of the first union of the rule, $C^{u2}$ and $D^{u2}$ are those of the second union, *etc*., and *A* is the action triggered by the rule (e.g. prescribing a given drug; actions are not detailed here for the sake of simplicity). These rules can also be written:

$$\begin{aligned}\begin{array}{cl} & \bigwedge \left\{ c\in C^{p}\right\} \\ \wedge & \bigwedge \left\{ d\in D^{p}\right\} \\ \wedge & \bigwedge \left\{ \lnot c\in C^{a}\right\} \\ \wedge & \bigwedge \left\{ \lnot d\in D^{a}\right\} \\ \wedge & \bigwedge _{z=1}^{\left| U\right| }\left( \bigvee \left\{ c\in C^{uz}\right\} \vee \bigvee \left\{ d\in D^{uz}\right\} \right) \,\rightarrow A \end{array} \end{aligned}$$

A clinical rule $R_{i}$ of that form can be formalized as a 6-element tuple $R_{i}=\left( C^{p},D^{p},C^{a},D^{a},U=\left\{ \left( C^{u1},D^{u1}\right) ,\left( C^{u2},D^{u2}\right) ,...\right\} ,A\right)$ where the first four elements are the sets of present and absent clinical and non-clinical conditions, the fifth element *U* is the set of the unions in the rule, each union being formalized as a pair of clinical and non-clinical conditions, and the sixth element is the action *A*.

For example, here are two clinical rules from STOPP/START:

- START rule D2: “Start fibre supplements (e.g. bran, ispaghula, methylcellulose, sterculia) for diverticulosis with a history of constipation”.

- STOPP rule D6: “Stop antipsychotics (*i.e.* other than quetiapine or clozapine) in those with parkinsonism or Lewy Body Disease (risk of severe extra-pyramidal symptoms)”.

They can be formalized as follows:

$$\begin{aligned} R_{D2}=constipation\wedge diverticulosis\wedge \lnot fibre\rightarrow start(fibre) \end{aligned}$$

$$\begin{aligned}\begin{array}{l}=\left( \begin{array}{l} C^{p}=\left\{ constipation,diverticulosis\right\} \\ D^{p}=\emptyset \\ C^{a}=\emptyset \\ D^{a}=\left\{ fibre\right\} \\ U=\emptyset \\ A=start(fibre) \end{array}\right) \end{array} \end{aligned}$$

$$\begin{aligned}\begin{array}{l} R_{D6}=antipsychotics\wedge (parkinsonism\vee Lewy\,\,Body)\\ \,\,\,\,\,\,\,\,\,\,\,\,\,\,\,\,\rightarrow stop(antipsychotics) \end{array} \end{aligned}$$

$$\begin{aligned}\begin{array}{l}=\left( \begin{array}{l} C^{p}=\emptyset \\ D^{p}=\left\{ antipsychotic\right\} \\ C^{a}=\emptyset \\ D^{a}=\emptyset \\ U=\left\{ \left( \left\{ parkinsonism,Lewy\,\,Body\right\} ,\emptyset \right) \right\} \\ A=stop(antipsychotic) \end{array}\right) \end{array} \end{aligned}$$

#### Display rule generation

We call *display rule* a rule that determines whether a given clinical condition *x* should be displayed and asked for manual entry by a clinician. Display rules are distinct from clinical rules, aiming at diagnosis or therapy. The action of a display rule is the display of a given clinical condition in the form. The set of conditions that should be displayed in the form can be obtained by executing all display rules and showing conditions for which at least one display rule states that it should be displayed. When the patient profile changes (e.g. a new drug is prescribed, or a clinical condition is entered), display rules must be executed again to determine the clinical conditions to display after the change.

In the design of the algorithm for generating display rules, we made the following assumptions: (a) It is preferable to display the lowest possible number of clinical conditions, in order to simplify the questionnaire and reduce the time required for data entry. (b) Non-clinical conditions (e.g. drug prescriptions or lab test results) are already known and coded (otherwise, they should be treated as clinical conditions in the following formula), and thus they will be checked first. (c) Clinical conditions are more likely to be false than true (this a “rule of thumb” that is verified for most conditions, e.g. les than 50% of patients have history of stroke, dementia, *etc.*; with the possible exception of hypertension and renal failure for the elderly). Thus, clinical conditions that must be present will be displayed before those that must be absent. For instance, for a rule requiring the presence of ankle edema and the absence of heart failure, we consider that it is better to ask first for the presence of ankle edema, and then, if present, to ask for the absence of heart failure, since the first condition (presence of ankle edema) has a lower chance of occurring.

Consequently, in display rules, the conditions will be considered in the following order of priority: first, check the $D^{p}$ and $D^{a}$ member of the rule 6-element tuple, then check $C^{a}$, finally check $C^{p}$ and *U*. For example, for $R_{D2}$, display rules will first check than fibre is absent ($D^{a}$), then display the first clinical condition (constipation or diverticulosis, $C^{p}$), and display the second one only if the first one is true. Whether constipation or diverticulosis is shown first is here an arbitrary choice, thus we have to define an order of priority between clinical conditions.

Let us consider a strict total order $\prec$ between the clinical conditions in *C*, such that $c1\prec c2$ means that *c*1 is displayed preferably than *c*2 when one of them must be chosen to be displayed first (e.g. if a rule has for conditions $c1\wedge c2$, and if $c1\prec c2$, then *c*1 is displayed, and *c*2 will be displayed only if *c*1 is present). We will explain later how to find the best order $\prec$. For each clinical condition $x\in C^{p}$ in rule $R_{i}$, we define the rule $\mathcal {R}^{p}\left( R_{i},x\right)$ that determines whether present clinical condition $x\in C^{p}$ needs to be displayed, or not, as follows:

$$\begin{aligned}\begin{array}{ccl} \mathcal {R}^{p}\left( R_{i},x\in C^{p}\right) & = & \bigwedge \left\{ c\in C^{p}\mid c\prec x\right\} \\ & \wedge & \bigwedge \left\{ d\in D^{p}\right\} \\ & \wedge & \bigwedge \left\{ \lnot c\in C^{a}\right\} \\ & \wedge & \bigwedge \left\{ \lnot d\in D^{a}\right\} \\ & \wedge & \bigwedge _{z=1}^{\left| U\right| }\left\{ \begin{array}{l} \bigvee \left\{ c\in C^{uz}\right\} \vee \bigvee \left\{ d\in D^{uz}\right\} \\ \,\,\,\,\,\,\,\,\,\,\,\,if\,\,\forall c\in C^{uz},c\prec x\\ True\,\,otherwise \end{array}\right. \\ & \rightarrow & display(x) \end{array} \end{aligned}$$

$\mathcal {R}^{p}$ displays the condition *x* if non-clinical conditions are satisfied ($D^{p}$ and $D^{a}$), absent clinical conditions are satisfied ($C^{a}$), and present clinical conditions in $C^{p}$ and *U* that have priority on *x* are satisfied.

We define two rules $\mathcal {R}^{a1}\left( R_{i},x\in C^{a}\right)$ and $\mathcal {R}^{a2}\left( R_{i},x\in C^{a}\right)$ for each clinical condition that must be absent:

$$\begin{aligned}\begin{array}{ccl} \mathcal {R}^{a1}\left( R_{i},x\in C^{a}\right) & = & \bigwedge \left\{ c\in C^{p}\right\} \\ & \wedge & \bigwedge \left\{ d\in D^{p}\right\} \\ & \wedge & \bigwedge \left\{ \lnot c\in C^{a}\mid c\ne x\right\} \\ & \wedge & \bigwedge \left\{ \lnot d\in D^{a}\right\} \\ & \wedge & \bigwedge _{z=1}^{\left| U\right| }\left( \bigvee \left\{ c\in C^{uz}\right\} \vee \bigvee \left\{ d\in D^{uz}\right\} \right) \\ & \rightarrow & display(x) \end{array}\end{aligned}$$

$$\begin{aligned}\begin{array}{ccl} \mathcal {R}^{a2}\left( R_{i},x\in C^{a}\right) & = & \left\{ x\right\} \\ & \wedge & \bigwedge \left\{ d\in D^{p}\right\} \\ & \wedge & \bigwedge \left\{ \lnot c\in C^{a}\mid c\prec x\right\} \\ & \wedge & \bigwedge \left\{ \lnot d\in D^{a}\right\} \\ & \rightarrow & display(x) \end{array} \end{aligned}$$

Since clinical conditions are expected to be more frequently false than true, absent conditions are checked lastly. $\mathcal {R}^{a1}$ displays the condition *x* if all other conditions are satisfied (*i.e.* non-clinical conditions $D^{p}$ and $D^{a}$, present clinical conditions $C^{p}$, unions *U* and other absent clinical conditions in $C^{a}$). $\mathcal {R}^{a2}$ displays the condition *x* if it is true and if other conditions that have priority are satisfied. $\mathcal {R}^{a2}$ is needed to permit the user to remove condition *x* if it has been set, but the other conditions of the rules have not been set. This normally cannot occur when interacting with the system starting from an empty list of clinical conditions; however, it may occur if the initial list of clinical conditions is not empty (e.g. if some clinical conditions are automatically extracted from EHR, and then the clinician verifies them and completes them is needed).

Finally, we define two rules $\mathcal {R}^{u1}\left( R_{i},x\in C^{uk}\right)$ and $\mathcal {R}^{u2}\left( R_{i},x\in C^{uk}\right)$ for each clinical condition that is a member of a union, with $1\le k\le \left| U\right|$.

$$\begin{aligned}\begin{array}{ccl} \mathcal {R}^{u1}\left( R_{i},x\in C^{uk}\right) & = & \bigwedge \left\{ c\in C^{p}\mid c\prec x\right\} \\ & \wedge & \bigwedge \left\{ d\in D^{p}\right\} \\ & \wedge & \bigwedge \left\{ \lnot c\in C^{a}\right\} \\ & \wedge & \bigwedge \left\{ \lnot d\in D^{a}\right\} \\ & \wedge & \bigwedge _{z=1}^{\left| U\right| }\left\{ \begin{array}{l} \bigwedge \left\{ \lnot c\in C^{uz}\right\} \wedge \bigwedge \left\{ \lnot d\in D^{uz}\right\} \\ \,\,\,\,\,\,\,\,\,\,\,\,if\,\,x\in C^{uz}\\ \bigvee \left\{ c\in C^{uz}\right\} \vee \bigvee \left\{ d\in D^{uz}\right\} \\ \,\,\,\,\,\,\,\,\,\,\,\,if\,\,\forall c\in C^{uz},c\prec x\\ True\,\,otherwise \end{array}\right. \\ & \rightarrow & display(x) \end{array} \end{aligned}$$

$$\begin{aligned}\begin{array}{ccl} \mathcal {R}^{u2}\left( R_{i},x\in C^{uk}\right) & = & \bigwedge \left\{ c\in C^{p}\mid c\prec x\right\} \\ & \wedge & \bigwedge \left\{ d\in D^{p}\right\} \\ & \wedge & \bigwedge \left\{ \lnot c\in C^{a}\right\} \\ & \wedge & \bigwedge \left\{ \lnot d\in D^{a}\right\} \\ & \wedge & \bigwedge _{z=1}^{\left| U\right| }\left\{ \begin{array}{l} \left\{ x\right\} \,\,\,\,if\,\,x\in C^{uz}\\ \bigvee \left\{ c\in C^{uz}\right\} \vee \bigvee \left\{ d\in D^{uz}\right\} \\ \,\,\,\,\,\,\,\,\,\,\,\,if\,\,\forall c\in C^{uz},c\prec x\\ True\,\,otherwise \end{array}\right. \\ & \rightarrow & display(x) \end{array} \end{aligned}$$

$\mathcal {R}^{u1}$ displays the condition *x* if no condition in the union is true and if other conditions that have priority are satisfied. $\mathcal {R}^{u2}$ displays the condition *x* if it is true and if other conditions that have priority are satisfied. Similarly to $\mathcal {R}^{a2}$, $\mathcal {R}^{u2}$ is only needed to permit the clinician to unset the condition *x* if it was set outside of the questionnaire.

Notice that display rules can be expressed as 6-element tuples, exactly as clinical rules. Considering $R_{i}=\left( C^{p},D^{p},C^{a},D^{a},U,A\right)$, we have:

$$\begin{aligned}\mathcal {R}^{p}\left( R_{i},x\in C^{p}\right) =\left( \begin{array}{l} \left\{ c\in C^{p}\mid c\prec x\right\} \\ D^{p}\\ C^{a}\\ D^{a}\\ \left\{ (C^{u},D^{u})\in U\mid \forall c\in C^{u},c\prec x\right\} \\ display(x) \end{array}\right) \end{aligned}$$

$$\begin{aligned}\mathcal {R}^{a1}\left( R_{i},x\in C^{a}\right) =\left( \begin{array}{l} C^{p}\\ D^{p}\\ C^{a}\setminus \left\{ x\right\} \\ D^{a}\\ U\\ display(x) \end{array}\right) \end{aligned}$$

$$\begin{aligned}\mathcal {R}^{a2}\left( R_{i},x\in C^{a}\right) =\left( \begin{array}{l} \left\{ x\right\} \\ D^{p}\\ \left\{ c\in C^{a}\mid c\prec x\right\} \\ D^{a}\\ \emptyset \\ display(x) \end{array}\right) \end{aligned}$$

$$\begin{aligned}\mathcal {R}^{u1}\left( R_{i},x\in C^{uk}\right) =\left( \begin{array}{l} \left\{ c\in C^{p}\mid c\prec x\right\} \\ D^{p}\\ C^{a}\cup C^{uk}\\ D^{a}\cup D^{uk}\\ \left\{ (C^{u},D^{u})\in U\mid C^{u}\ne C^{uk}\wedge \forall c\in C^{u},c\prec x\right\} \\ display(x) \end{array}\right) \end{aligned}$$

$$\begin{aligned}\mathcal {R}^{u2}\left( R_{i},x\in C^{uk}\right) =\left( \begin{array}{l} \left\{ c\in C^{p}\mid c\prec x\right\} \cup \left\{ x\right\} \\ D^{p}\\ C^{a}\\ D^{a}\\ \left\{ (C^{u},D^{u})\in U\mid C^{u}\ne C^{uk}\wedge \forall c\in C^{u},c\prec x\right\} \\ display(x) \end{array}\right) \end{aligned}$$

We denote by $R^{d}$ the set of all display rules generated from the clinical rules in *R*.

For example, for the START rule D2 and STOPP rule D6 above, and considering $constipation\prec diverticulosis$, we obtain the following display rules:

$$\begin{aligned}\begin{array}{l} \mathcal {R}^{p}\left( R_{D2},constipation\right) =\lnot fibre\\ \,\,\,\,\,\,\,\,\,\,\,\,\,\,\,\,\,\,\,\,\,\,\,\,\,\,\,\,\,\,\,\,\,\,\,\,\,\,\,\,\,\,\,\,\,\,\,\,\,\,\,\,\,\,\,\,\,\,\,\,\,\rightarrow display(constipation) \end{array} \end{aligned}$$

$$\begin{aligned} \begin{array}{l} \mathcal {R}^{p}\left( R_{D2},diverticulosis\right) =constipation\wedge \lnot fibre\\ \,\,\,\,\,\,\,\,\,\,\,\,\,\,\,\,\,\,\,\,\,\,\,\,\,\,\,\,\,\,\,\,\,\,\,\,\,\,\,\,\,\,\,\,\,\,\,\,\,\,\,\,\,\,\,\,\,\,\,\,\,\,\,\,\rightarrow display(diverticulosis) \end{array} \end{aligned}$$

$$\begin{aligned}\begin{array}{l} \mathcal {R}^{u1}\left( R_{D6},parkinsonism\right) =antipsychotics\wedge \lnot Lewy\,\,Body\\ \,\,\,\,\,\,\,\,\,\,\,\,\,\,\,\,\,\,\,\,\,\,\,\,\,\,\,\,\,\,\,\,\,\,\,\,\,\,\,\,\,\,\,\,\,\,\,\,\,\,\,\,\,\,\,\,\,\,\,\,\,\,\,\,\rightarrow display(parkinsonism) \end{array} \end{aligned}$$

$$\begin{aligned}\begin{array}{l} \mathcal {R}^{u2}\left( R_{D6},parkinsonism\right) =antipsychotics\wedge parkinsonism\\ \,\,\,\,\,\,\,\,\,\,\,\,\,\,\,\,\,\,\,\,\,\,\,\,\,\,\,\,\,\,\,\,\,\,\,\,\,\,\,\,\,\,\,\,\,\,\,\,\,\,\,\,\,\,\,\,\,\,\,\,\,\,\,\,\rightarrow display(parkinsonism) \end{array} \end{aligned}$$

$$\begin{aligned}\begin{array}{l} \mathcal {R}^{u1}\left( R_{D6},Lewy\,\,Body\right) =antipsychotics\wedge \lnot parkinsonism\\ \,\,\,\,\,\,\,\,\,\,\,\,\,\,\,\,\,\,\,\,\,\,\,\,\,\,\,\,\,\,\,\,\,\,\,\,\,\,\,\,\,\,\,\,\,\,\,\,\,\,\,\,\,\,\,\,\,\,\,\rightarrow display(Lewy\,\,Body) \end{array} \end{aligned}$$

$$\begin{aligned}\begin{array}{l} \mathcal {R}^{u2}\left( R_{D6},Lewy\,\,Body\right) =antipsychotics\wedge Lewy\,\,Body\\ \,\,\,\,\,\,\,\,\,\,\,\,\,\,\,\,\,\,\,\,\,\,\,\,\,\,\,\,\,\,\,\,\,\,\,\,\,\,\,\,\,\,\,\,\,\,\,\,\,\,\,\,\,\,\,\,\,\,\,\rightarrow display(Lewy\,\,Body) \end{array} \end{aligned}$$

For START rule D2, if fibre is already prescribed, no clinical condition is displayed. Otherwise, only constipation is displayed. When constipation is checked, diverticulosis is displayed.

#### Formalization of the ordering problem

Identifying the best global strategy for asking clinical conditions can be formalized as an ordering problem: finding the optimal strict total order $\prec$ between the clinical conditions in *C* that minimizes the number of distinct clinical conditions displayed in the questionnaire when no clinical conditions are entered yet, *i.e.* finding $\prec$ that minimizes:

$$\begin{aligned}\begin{array}{l} \Big |\Big \{ x\in C\mid \exists R_{j}^{d}\in R^{d}\,\,such\,\,as\,\,R_{j}^{d}=conditions\rightarrow display(x)\\ \,\,\,\,\,\,\,\,\,\,\,\,\,\,\,\,\,\,\,\,\,\,\,\,\,\,\,\,\,\,\,\,\,\,\,\,\,\,\,\,\,\,\,\,\,\,\,\,\,\,\,\,\,\,\,\,\,\,\,\,\,\,\,\,\,\,\,and\,\,conditions\,\,are\,\,satisfied\Big \}\Big | \end{array} \end{aligned}$$

#### Proof of NP-hardness

The ordering problem can be proved to be NP-hard, by reducing it to the Generalized Traveling Salesman Problem (GTSP). The Traveling Salesman Problem (TSP) consists in finding the shortest travel that passes through a given set of towns. The GTSP is similar, but also considers a set of areas, each town being located in a given area, and the travel must pass through one town of each area (instead of all towns). Both TSP and GTSP are NP-hard.

For the sake of simplicity, we will restrict the proof to simpler display rules of the form $R'=\bigwedge \left\{ c\in C^{p}\right\} \rightarrow A=\left( C^{p},\emptyset ,\emptyset ,\emptyset ,\emptyset ,A\right)$, and we will consider that all patient conditions are false. With such rules, the order $\prec$ can be built progressively, from left to right. An order $\prec$ under construction is of the form $C_{a}\prec C_{b}\prec ...\prec C_{\alpha }\simeq C_{\beta }\simeq ...$. In such order, conditions $C_{ord}=\left\{ C_{a},C_{b},...\right\}$ have been ordered and other conditions $C_{unord}=\left\{ C_{\alpha },C_{\beta },...\right\}$ have not yet been ordered, with $\forall c\in C_{ord}\forall c'\in C_{unord},c\prec c'$. In the definition of $\mathcal {R}^{p}(R_{i},x)$, if we consider a rule of the form $R'$, we can see that it will generate display rules of the form: $True\rightarrow display(C_{a})$, $C_{a}\rightarrow display(C_{b})$, $C_{a}\wedge C_{b}\rightarrow display(C_{c})$,... for the order $C_{a}\prec C_{b}\prec C_{c}\prec ...$. If we assume that, in the initial form, all clinical conditions are false, the display rules $\mathcal {R}^{p}(R_{i},x)$ will lead to the display of a single condition, the first one according to the order $\prec$ in $C^{p}$. Let us note $C_{r}^{p}$ the $C^{p}$ component of the rule *r*. We search the order $\prec$ that minimizes the number of clinical conditions displayed in the initial form, *i.e.*:

$$\begin{aligned} \left| \left\{ x\mid \exists r\in R^{d}\,\,such\,\,that\,\,x\in C_{r}^{p}\,\,and\,\,\forall y\in C_{r}^{p}\,\,with\,\,y\ne x,x\prec y\right\} \right| \end{aligned}$$

In the GTSP reformulation of the problem, the distance corresponds to the number of clinical conditions displayed in the form. We cannot represent each clinical condition by a town. In fact, in GTSP, when adding a new town in the travel, the cost (*i.e.* the distance) depends only on the current town and the new town added; on the contrary, in our problem, when adding a new clinical condition *x* to the right of the order $\prec$ , the cost depends on all clinical conditions already present in the order $\prec$ under construction, and not only on the last one. The cost of the addition is 1 (*i.e.*, one new condition to display in the form) if there is at least one rule that includes *x* in its conditions $C^{p}$ and that includes no other condition already present in the order $\prec$ under construction; otherwise it is 0 (no new condition to display).

Consequently, we considered a town as being the subset of the clinical conditions currently included in the order $\prec$ under construction (*i.e.* $C_{ord}$). Thus, the ordering problem can be rewritten as a GTSP by considering a set of $2^{n}$ towns $T=\left\{ x\subseteq C\right\}$, and a set of $n+1$ areas $A=\left\{ A_{0},A_{1},...,A_{n}\right\}$, each town *t* belonging to the area $A_{\left| t\right| }$, *i.e.* $A_{k}=\left\{ t\in T\mid \left| t\right| =k\right\}$.

The optimal strict total order can be deduced by ordering the clinical conditions in their order of appearance in the town sets. For example, if the solution found is $\left( \left\{ \right\} ,\left\{ C_{1}\right\} ,\left\{ C_{1},C_{3}\right\} ,\left\{ C_{1},C_{3},C_{2}\right\} \right)$, then the optimal total order is $C_{1}\prec C_{3}\prec C_{2}$.

The asymmetric distance matrix *M* of the GTSP is defined as follows:

$$\begin{aligned} M(i\in T,j\in T)=\left\{ \begin{array}{ll} 0 & if\,\,i\in A_{n}\wedge j\in A_{0}\\ +\infty & if\,\,\lnot \left( i\in A_{k}\wedge j\in A_{k+1}\right) \\ 1 & \exists r\in R\mid j\setminus i\subseteq C_{r}^{p}\wedge i\cap C_{r}^{p}=\emptyset \\ 0 & otherwise \end{array}\right. \end{aligned}$$

The first condition gives a distance of 0 for closing the loop of the travel.

The second condition gives a distance of $+\infty$ when the salesman travels from a town in the area $A_{k}$ to a town that is not in area $A_{k+1}$, in order to force to visit all areas in order.

The third condition gives a distance of 1 if the salesman travels to a town that adds a new clinical condition that is present in a rule for which no other condition is already present in the departure town.

The distance is 0 otherwise.

#### Solving the ordering problem

NP-hard problems can only be solved by testing all possible solutions, or by using heuristic algorithms that give a good solution, but not necessarily the best one. In theory, the optimal clinical conditions order is patient dependent: in particular, rules having non-clinical conditions only impact patients having these conditions.

We considered two options for solving the ordering problem: (1) a simple heuristic algorithm that sorts clinical conditions in decreasing order of their number of occurrences in the rules, producing a global, patient independent, order, and (2) the Artificial Feeding Birds (AFB) metaheuristic [39], for computing a near optimal, patient-specific order.

### User interface design

We designed a user interface for displaying the adaptive questionnaire, following agile software development methods and relying on prototyping using web technologies (HTML, CSS and Brython, a Javascript-compiled version of Python).

Each clinical condition is displayed as a checkbox in the user interface. Checkboxes are grouped in 13 general categories (e.g. cardiology, digestive system, *etc.*) to facilitate the search of a particular condition. We previously designed and used these 13 categories and their associated colors in a work focused on adverse drug events [40].

Some conditions are associated with several codes in the terminology, e.g. “diabetes” can be associated with ICD10 codes E10 (insulin-dependent diabetes mellitus), E11 (non-insulin-dependent diabetes mellitus), *etc*. In that case, when the box is checked, a drop-down combo box allows the user to select the appropriate term. By default, the most general term is selected, e.g. E14 (unspecified diabetes mellitus).

In rare cases, a clinical condition may be more general than another one, *i.e.* related together with “is a” relation (e.g. “diabetes” is more general than “type 2 diabetes”). In that case, only the most general condition is displayed (e.g. “diabetes”), and the user may select the desired code in the drop-down combo box (including the code for the more specific condition).

When new conditions appear during user interaction, following the selection of a given condition, the new conditions are highlighted in yellow and marked with a red star.

### Evaluation methods

#### Test on clinical cases

In a first experiment, the proposed method was tested on 10 realistic clinical cases designed by a GP (HF, available as Supplementary file #2). We evaluated both the number of clinical conditions displayed and the time required for executing the display rules. The method was executed on a modern laptop computer (processor: Intel Core i7-10510U CPU, 1.80 GHz).

#### Test on real patients from Rouen hospital

In a second experiment, the proposed method was tested on retrospective anonymized data from about one hundred patients extracted from the Rouen hospital, France and the EDSaN (*Entrepôt de Données de Santé Normand*, Normandy clinical data warehouse) [41]. For this experiment, ethics approval was obtained from the ethic comity of Rouen hospital. The patients were randomly chosen in the database, among patients taking 5+ drugs and being 65+ years old. We used the following structured data available in the clinical data warehouse: patient sex and birth date, clinical conditions (*i.e.* ICD10 codes), drug orders (French UCD code, *Unités Communes de dispensation*) and lab test results (LOINC codes). We evaluated the number of clinical conditions displayed.

#### Focus groups

In a third experiment, we organized focus group sessions for presenting ABiMed, our CDSS for medication reviews, to clinicians (including both GPs and pharmacists). A prototype of our CDSS was shown, various user interfaces focused on polypharmacy management were presented during the focus groups, including the proposed adaptive questionnaire and the implementation of the STOPP/START v2 guideline. Then, a clinical case was given and solved together, using the support of the CDSS. We asked the opinion of the clinicians, and in particular whether the fact that checkboxes may appear or disappear during the data entry would be disturbing, or not.

The recruitment period was from 1/5/2022 to 31/6/2022. Ethics approval is not required in France for the type of study we conducted: health professionals were simply presented a new software and they gave their opinion on it. No real patient data were involved, and no personal data was collected, everything remaining fully anonymous. Informed consent is assumed to be implicit, the active participation to the study being considered as a consent, according to the French regulation.

## Results

### Adaptive questionnaire for STOPP/START v2

The proposed methods were applied to STOPP/START v2. Three recommendations were considered as too general for implementation (STOPP A1, A2 and A3). The other 111 recommendations were translated into 124 clinical rules (a few recommendations were split in two rules). The implementation of STOPP/START v2 considered a total of 73 distinct clinical conditions (55 for STOPP rules and 30 for START, 12 conditions belonging to both). Using the proposed methods, 197 display rules were generated (136 for STOPP rules and 61 for START).

**Fig. 1:**
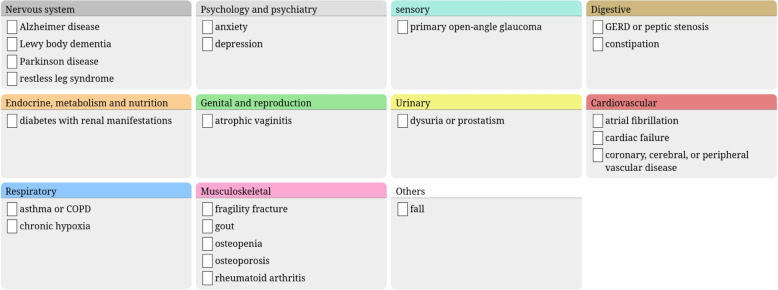
The adaptive questionnaire for a patient having an empty drug order

**Fig. 2:**
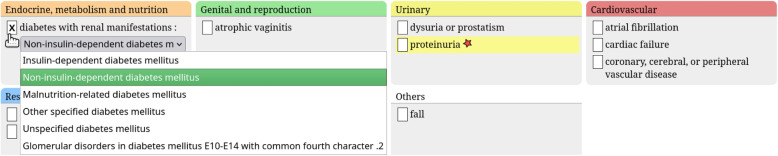
Excerpt of the adaptive questionnaire of Fig. 1, after the user checked the condition “diabetes with renal manifestations”. Notice the drop-down combo box for choosing the appropriate ICD10 term, and the new condition “proteinuria” that appeared on the right

### User interface

Figure 1 shows the adaptive questionnaire we designed, for a patient having an empty drug order. There are 23 conditions displayed, grouped in anatomical categories. Each category is represented by a panel with a colored title bar, and each condition is represented by a checkbox. Panels are organized on 4 columns, in order to display all conditions on a single screen, without having to scroll up and down.

As a matter of comparison, supplementary file #1 shows the entire questionnaire, if it was not adaptive, thus displaying all 73 clinical conditions.

Figure 2 shows the same questionnaire after the user checked the “diabetes with renal manifestations” condition. In addition to the check in the checkbox, the questionnaire was adapted on two points. First, a drop-down combo box is now displayed for choosing the appropriate ICD10 term (since there are several ICD10 terms that correspond to diabetes with renal manifestations). Second, a new condition, “proteinuria”, appeared on the right. This condition was not necessary for applying STOPP/START rules before checking “diabetes with renal manifestations”, but becomes necessary after. The new condition is temporarily highlighted with a yellow background, to attract user attention. It also has a red star after its label. The red star is more subtle than the yellow background, but remains permanently.

Similarly, conditions may disappear during user interaction, e.g. the “proteinuria” condition would disappear if the user unchecks the “diabetes with renal manifestations” condition.

**Table 1:** The top part of the table shows the results obtained when testing the proposed method on 10 clinical cases. Execution time for display rules, number of conditions displayed and number of rules triggered. The bottom part shows the aggregated results obtained when testing on real patient data. ^1^: the total of conditions displayed may be less than the sum of STOPP and START (e.g. case #1) , because some conditions are common

				**Conditions displayed**			**Rules triggered**		
	**Case**	**# drugs**	**Time (s)**	STOPP (55)	START (30)	Total^1^ (73)	STOPP (77)	START (34)	Total (111)
Clinical cases	#1	11	0.075	11	21	28	5	3	8
	#2	12	0.065	6	22	27	0	6	6
	#3	15	0.061	5	17	20	4	4	8
	#4	9	0.058	7	18	24	3	5	8
	#5	6	0.057	4	20	24	2	3	5
	#6	11	0.064	16	20	29	8	6	14
	#7	13	0.072	6	20	25	3	4	7
	#8	15	0.066	10	19	29	3	3	6
	#9	10	0.061	13	22	34	4	2	6
	#10	13	0.060	8	20	27	1	4	5
	**Mean**	11.5	0.064	8.6	19.9	**26.7**	3.3	4.0	7.3
	**(%)**			(15.6%)	(66.3%)	**(36.6%)**			
Real data	**Mean**			6.4	21.5	**25.5**	3.5	4.2	7.7
	**(%)**			(11.6%)	(71.7%)	**(35.0%)**			
	Min.			0	16	19	0	1	1
	Max.			17	25	34	15	9	21
	Std.			3.1	1.8	2.7	3.0	1.7	3.7

### Evaluation results

#### Test on clinical cases

Table 1 (top) shows the results obtained when applying the proposed methods to 10 realistic clinical cases of old patients with polypharmacy, using the simple heuristic algorithm described in Section Solving the ordering problem for finding a global order of the clinical conditions. The execution time remains lower than 0.1 second on a modern computer, which is compatible with a clinical use. When using the AFB metaheuristic to compute a patient-specific clinical conditions order, the algorithm quickly converged, and we obtained exactly the same results in terms of the number of conditions displayed, but the execution time was longer (about 0.4 second). We thus keep the simple heuristic algorithm.

Out of the 73 clinical conditions required for STOPP/START v2, only 26.7 conditions are displayed on average, which is 36.6% (almost a two-third reduction). This represents an important reduction in the number of conditions to enter or review manually. More specifically, the reduction is much more important for STOPP (only 15.6% of the conditions are displayed), than for START (66.3% of the conditions are displayed). This was expected, because all STOPP recommendations have a drug condition that must be present (*i.e.* $D^{p}\ne \emptyset$), thus allowing displaying the clinical conditions of the recommendation only if the drug is present. On the contrary, START recommendations may have no such drug condition. Nevertheless, we observe that the proposed method still substantially reduces the number of conditions displayed for START.

#### Test on real patients from Rouen hospital

We extracted retrospective real data for 119 patients. Table 1 (bottom) shows the aggregated results obtained when applying the proposed methods to the real patient data. These results show that only 25.5 conditions are displayed on average, which is 35.0% of the total number of conditions mentioned in STOPP/START v2. As above, the reduction is more important for STOPP than for START, for the same reasons. The results on real data are similar to the ones obtained on clinical cases.

#### Focus groups

We organized two focus group sessions, with a total of 16 clinicians (8 GPs and 8 pharmacists). The first session was performed in a rural environment, with 4 pharmacists (2 males, 2 females) and 4 GPs (4 males); 7 clinicians were young (30-40) and 1 was older (> 65). The second session was performed in a city environment, with 4 pharmacists (3 males, 1 females) and 4 GPs (2 males, 2 females); 4 were young (30-40) and 4 were older (50-65).

Clinicians understood easily how the adaptive questionnaire was working, and its interest for reducing and simplifying data entry. They found it easy to use and potentially useful for gaining time. Clinicians said that “it was pretty easy to use”, “visually simple” and that “really we had just the data we needed in fact in the adaptive questionnaire, really everything that was a bit polluting for us at the level of the screen did not appear”. Clinicians appreciated having automatic data extraction from the EHR when possible, but also the possibility to manually review and complement the data in the adaptive questionnaire, or enter it from scratch when data extraction is not possible (e.g. if the GP uses an EHR that is not compatible, or does not agree on data transfers). More generally, all clinicians agreed that the CDSS will be useful to them and that the interfaces were appropriate.

## Discussion

In this paper, we designed an adaptive questionnaire for facilitating patient condition entry in a clinical decision support system for medication reviews implementing STOPP/START v2. We showed that this approach is able to reduce by almost two thirds the length of the questionnaire.

Rule-based systems are not a novel technique for clinical decision support. Nevertheless, they are still widely used for implementing clinical practice guidelines, especially for therapeutics. Our rule-based system implementing STOPP/START v2 is not innovative in itself, however, the data entry method we proposed, relying on an adaptive questionnaire automatically generated from the translation of the rules of the system, is a novel approach for facilitating clinical data entry in a clinical decision support system.

We made three assumptions when generating the display rules (see Section Display rule generation): (a) it is preferable to display fewer clinical conditions in the questionnaire, (b) drug prescriptions and lab test results are already known and coded and (c) clinical conditions are more likely to be absent than present. Assumption (a) is sounding since our objective is to simplify the questionnaire and reduce its size. Assumption (b) assumes that drug data is already available. This is usually the case in community pharmacy, and, anyway, one of the first steps in the medication review is to list all drugs taken by the patient. Nevertheless, if prescriptions would not be available, the method can easily be adapted by treating them in formulas as we treated clinical conditions. Additionally, for STOPP/START, lab test conditions match clinical conditions, e.g. “serum K+ < 3.0 mmol/l” matches hypokalemia, therefore, lab test conditions are not needed in the questionnaire (but they are still important to have in the clinical rules, in case of coded lab test results could be extracted). Assumption (c) holds for most clinical conditions, and also corresponds to the usual way of thinking of clinicians: by default, a patient is considered as not having a given disorder unless it is mentioned in his record. As a perspective, a more sophisticated system may allow a manual prioritization of the clinical conditions, whenever needed for a specific rule.

We compared two methods for ordering the clinical conditions: a simple heuristic algorithm and a metaheuristic (AFB). Both yielded the same results in terms of the number of conditions displayed, despite the metaheuristic is more sophisticated and can, in theory, provide a better, patient-specific, ordering leading to a shorter questionnaire. This can probably be explained by the fact that the ordering problem in theoretically NP-hard, but in practice, many clinical conditions are unrelated and independent from each other, at least in the STOPP/START v2 guideline. As a consequence, there is no interest in using the metaheuristic, which is computationally much more expensive, over the simple heuristic algorithm for STOPP/START v2. However, this might not be the case with other guidelines, thus having a more sophisticated option for solving the ordering problem remains interesting.

We applied the proposed method to STOPP/START v2, a guideline for managing polypharmacy. This guideline is particularly favorable to the use of adaptive questionnaires, because all STOPP rules have at least one non-clinical condition (*i.e.* a drug prescription). The application of our method to another guideline aimed at the treatment of a given disorder, such as type 2 diabetes or hypertension, is expected to raise some challenges and require some adaptation. First, the reduction of the number of conditions displayed is expected to be lower than the one observed on STOPP/START, probably similar to the one observed on START rules only (*i.e.* about one third). Second, the application to a new guideline would require to formalize that guidelines into if-then rules following the format described in Section Clinical rule formalization. This model is generic and should match most rules; however, some specific rules may not match that model and would require an extension of our system. An example we encountered in cardiovascular guidelines in other projects is a rule of the form “if at least *x* conditions are present in a given list of *y* conditions, then...”. Third, the ordering problem described above needs to be assessed for the new application, and in particular studying whether the simple heuristic algorithm we provided is sufficient, or more complex metaheuristic needs to be used. Fourth, the interface for presenting the adaptive questionnaire may need to be adapted to the new application. For instance, the classification of clinical condition in anatomical categories may not be appropriate for a cardiovascular guideline in which most conditions belong to the cardiovascular category. Finally, the new application would, of course, require careful evaluation in the appropriate clinical context.

The results on clinical cases and on real patient data show a reduction of the number of clinical conditions by almost two thirds. That reduction could make the difference between a questionnaire judged as “too long” by clinicians and a questionnaire that fit in a single screen (no scroll bar) and is usable in practices. Clinical cases and real patient data yield similar results. This increases the confidence in the results, but also confirms that our clinical cases are realistic.

In the literature, many CDSS for polypharmacy rely on the manual entry of clinical conditions from a medical terminology or a thesaurus : in a recent review [8], we found that only 7 out of 19 systems were connected to the EHR, most of them targeting hospital and not primary care. Examples of systems not connected to the EHR are: PIM-Check [42], KALIS [20] and PRIMA-eDS [43], while examples of connected systems are: STRIPA [44], TRIM [45] and MedSafer [19]. For manual data entry, PIM-Check uses a flat list of about 150 terms. PRIMA-eDS also uses a flat list. In the PRIMA-eDS evaluation, the users explicitly reported that the manual data entry was “inconvenient and time-consuming”, preventing the use of the tool in daily practice [43]. These studies focused on rule execution and validation in clinical situation, but no on data entry. It is only recently that usability has been considered for medication review support tools [46]. The other studies relied on EHR data extraction, however, the connection to EHR is usually difficult to achieve, especially for community pharmacists : EHR are located at hospital or at the GP office, and not available to community pharmacists. In practice, EHR connection was achieved only in hospital or for GPs, with a single exception for pharmacists in the Veterans Affairs in US, which is a very specific and centralized situations, as it operates centers integrating GPs and pharmacists (for TRIM [45]). But CDSS for polypharmacy are of particular use for community pharmacists when they perform medication reviews, and no solution has been proposed yet for facilitating the manual data entry in polypharmacy CDSS by the pharmacist, who usually do not have access to EHR.

The main limitation of this study is that, in most contexts other than polypharmacy, CDSS are used by physicians and not by pharmacists, and thus the users have access to the patient data in the EHR. However, the proposed adaptive questionnaire remains interesting as a tool for verifying at a glance the absence of error and missing data in the extracted patient data, before running the CDSS, or when the CDSS require very specific or trivial data that is unlikely to be present in the EHR. We encountered such a situation recently when designing a CDSS for the diagnosis and management of Covid-19 patients [47]: the CDSS required many symptoms (e.g. cough, rhinorrhea,...) which are usually not entered in the EHR.

Another limitation is that our questionnaire asks medical data to the pharmacist, but we did not study whether the pharmacist has access to all the required information when working in community. In most cases, the pharmacist could ask the patient, but this may be problematic in some situations (e.g. if the patient does not speak the same language). In real clinical conditions, the use of a CDSS is often complicated by the lack of time that the clinicians can dedicate to the system. However, medication review is a long task (more than one hour) that commonly performed by pharmacists in two stages: the interview with the patient for collecting data, and the analysis of the drug order, performed without the patient. The system we propose in this paper could be used at both stages: during patient interview for guiding the questions of the pharmacist towards the clinical elements that may trigger STOPP/START rules, and during analysis in the absence of the patient for orienting the eventual question that the pharmacist may ask to the GP.

The use of CDSS may also raise ethical problems and create biases [2]. As patient data is used by the CDSS the data may leak, raising privacy issues. Machine-learning-based CDSS are associated with additional privacy-related risks, since the patient data used for learning might be exposed. Automation bias is the propensity of people to over rely on a CDSS [48]. However, over-reliance may lead to the wrong decision when the CDSS recommendations are not appropriate, e.g. if the patient data used by the CDSS is not accurate. Contrary to automatic data extraction from EHR, our adaptive questionnaire allows clinicians to manually review the data, potentially preventing errors. Alert fatigue is another typical risk of CDSS, e.g. pharmacists may start ignoring alerts such as STOPP/START rules. Further studies are needed to evaluate this point.

The perspectives of this work include the evaluation of the STOPP/START v2 implementation in clinical practices during decision support, including more detailed feedback on usability and workflow integration, and the application of the adaptive questionnaire to other clinical practice guidelines. Our perspectives also include upgrading our CDSS to the recent STOPP/START v3. The method may also be extended to more complex rule format, for instance rules of the form “if at least *x* conditions are present in a given list of *y* conditions, then...”. Finally, adaptive questionnaires might also be of interest for collecting patient-reported outcomes and more generally for data entry by the patient itself.

## Conclusion

In conclusion, we proposed a method based on adaptive questionnaires for facilitating data entry in a clinical decision support system for medication reviews that implements STOPP/START v2, a clinical practice guideline for the management of polypharmacy. The method considers a guideline implemented as a rule-based system, and proposes formulas for translating the rules formalized from the guideline into rules determining which clinical conditions are mandatory to display to the clinician. Our tests on STOPP/START v2 showed that this method can reduce by almost two thirds the size of the questionnaire, for both clinical cases and real patient data. From a technical point of view, this method could be applied to any other CDSS that use rules of a similar format, and the formulas we propose could be extended for more complex rules. It can be of interest for verifying and completing the patient data extracted from EHR before executing the CDSS, or when automatic data extraction from the EHR is not possible. The use of adaptive questionnaires in CDSS could facilitate the data entry and thus improve CDSS acceptance by clinicians; it represents an improvement over the usual fixed questionnaires.

## Supplementary information

Supplementary Material 1

Supplementary Material 2

## Data Availability

The clinical cases used during the current study is available in this published article’s supplementary files.

## Abbreviations

CDSS: Clinical Decision Support System
EHR: Electronic Health Record
GP: General Practitioner
ICD10: International Classification of Diseases, release 10
LOINC: Logical Observation Identifiers Names & Codes
SPARQL: SPARQL Protocol and RDF Query Language
TSP: Traveling Salesman Problem
