# Correction: Adaptive questionnaires for facilitating patient data entry in clinical decision support systems: methods and application to STOPP/ START v2

**DOI:** 10.1186/s12911-025-03218-x

**Published:** 2025-10-02

**Authors:** Jean-Baptiste Lamy, Abdelmalek Mouazer, Romain Léguillon, Romain Lelong, Stéfan Darmoni, Karima Sedki, Sophie Dubois, Hector Falcoff

**Affiliations:** 1 INSERM, Sorbonne Universite, Universite Sorbonne Paris Nord, Laboratory of Medical Informatics and Knowledge Engineering in e-Health, LIMICS, 15 rue de l’école de médecine, Paris, 75006 France; 2 Department of Biomedical Informatics, Rouen University Hospital, Rouen, 76000 France; 3 SFTG Recherche (Société de Formation Thérapeutique du Généraliste), Paris, 75013 France

**Correction to: Jean-Baptiste et al. BMC Medical Informatics and Decision Making (2024) 24:326**

10.1186/s12911-024-02742-6

After the original article was published, the authors reported that all given and family names of the authors had been mistakenly reversed. The corrected names provided in this Correction are accurate.

The original article has been corrected.

