# Supplementary material for: Adaptive questionnaires for facilitating patient data entry in clinical decision support systems: methods and application to STOPP/START v2
Source: BMC Med Inform Decis Mak. 2024 Nov 5;24:326. doi: 10.1186/s12911-024-02742-6 (PMC11539734; doi:10.1186/s12911-024-02742-6)
Supplement: Supplementary file 1 — Supplementary Material 1 [file 12911_2024_2742_MOESM1_ESM.pdf]

Supplementary file #1

June 4, 2024

Screenshot of the entire questionnaire, with the "adaptive" functionality disabled.
It displays all clinical conditions for STOPP/START v2.

Nervous system

- [ ] Alzheimer disease
- [ ] Lewy body dementia
- [ ] Parkinson disease
- [ ] benign essential tremors
- [ ] dementia
- [ ] extrapyramidal symptoms
- [ ] parkinsonian syndrom
- [ ] restless leg syndrome

Psychology and psychiatry

- [ ] anxiety
- [ ] confusional syndrome
- [ ] depression
- [ ] psychosis
- [ ] sleep disorder

sensory

- [ ] acute angle glaucoma
- [ ] primary open-angle glaucoma

Digestive

- [ ] GERD or peptic stenosis
- [ ] constipation
- [ ] diverticulosis
- [ ] gastroduodenal ulcer
- [ ] gastrointestinal bleeding
- [ ] non complicated gastroduodenal ulcer
- [ ] peptic esophagitis
- [ ] upper digestive tract disorder

Endocrine, metabolism and nutrition

- [ ] diabetes
- [ ] diabetes with renal manifestations
- [ ] hepatic failure
- [ ] hypercalcemia
- [ ] hyperkaliemia
- [ ] hypoglycemia
- [ ] hypokaliemia
- [ ] hyponatremia
- [ ] iron deficiency
- [ ] type 2 diabetes

Genital and reproduction

- [ ] atrophic vaginitis
- [ ] breast cancer
- [ ] hypogonadism

Urinary

- [ ] chronic renal failure
- [ ] dysuria or prostatism
- [ ] nephrotic syndrom
- [ ] proteinuria
- [ ] renal failure
- [ ] urinary incontinence

Cardiovascular

- [ ] atrial fibrillation
- [ ] atrioventricular block
- [ ] bleeding risk
- [ ] bradycardia
- [ ] cardiac conduction disorder
- [ ] cardiac failure
- [ ] class III or IV cardiac failure
- [ ] coronary disease
- [ ] coronary disease
- [ ] coronary stent
- [ ] coronary, cerebral, or peripheral vascular disease
- [ ] deep vein thrombosis
- [ ] hypertension
- [ ] lower limb edema
- [ ] orthostatic hypotension
- [ ] pulmonary embolism
- [ ] supraventricular tachycardia
- [ ] venous thromboembolism

Respiratory

- [ ] COPD
- [ ] acute respiratory failure
- [ ] asthma or COPD
- [ ] chronic hypoxia

Musculoskeletal

- [ ] arthrosis
- [ ] fragility fracture
- [ ] gout
- [ ] osteopenia
- [ ] osteoporosis
- [ ] rheumatoid arthritis

Others

- [ ] fall
- [ ] pain
- [ ] syncope
